# Evaluating the Role of Food Security in the Context of Quality of Life in Underserved Communities: The ISAC Approach

**DOI:** 10.3390/nu17152521

**Published:** 2025-07-31

**Authors:** Terrence W. Thomas, Murat Cankurt

**Affiliations:** Department of Agribusiness, Applied Economics and Agriscience Education, North Carolina Agricultural and Technical State University, Greensboro, NC 27411, USA; mcankurt@ncat.edu

**Keywords:** food security, sustainable food system, quality of life, ISAC analysis procedure, best-worst scaling, underserved communities

## Abstract

**Background/Objectives:** Quality of life (QOL) is a multifaceted concept involving a variety of factors which define the overall well-being of individuals. Food security, which implies a resilient food system, is one factor that is central to the calculus of the QOL status of a community considering that food is a staple of life. Advancing food security as a strategy for attaining sustained improvement in community QOL hinges on recognizing that food security is embedded in a matrix of other factors that work with it to generate the QOL the community experiences. The lived experience of the community defines the community’s QOL value matrix and the relative position of food security in that value matrix. Our thesis is that the role of food security in the lived experience of low-income communities depends on the position food security is accorded relative to other factors in the QOL value matrix of the community. **Methods:** This study employed a multimethod approach to define the QOL value matrix of low-income Guilford County residents, identifying the relative position of the value components and demographic segments based on priority ranking. First, an in-depth interview was conducted and then a telephone survey (280 sample) was used for collecting data. The ISAC Analysis Procedure and Best–Worst Scaling methods were used to identify and rank components of the QOL value matrix in terms of their relative impact on QOL. **Results:** The analysis revealed that spiritual well-being is the most important contributor to QOL, with a weight of 0.23, followed by access to health services (0.21) and economic opportunities (0.16), while food security has a moderate impact with 0.07. **Conclusions:** These findings emphasize the need for targeted policy interventions that consider the specific needs of different demographic segments to effectively improve QOL and inform the design of resilient food systems that reflect the lived experiences of low-income communities. Food security policies must be integrated with broader quality of life interventions, particularly for unemployed, low-educated, and single individuals, to ensure that a resilient food system effectively reduces inequities and address community-specific vulnerabilities.

## 1. Introduction

It is widely agreed that sustainability is a concept that refers to the ability to conduct socioeconomic activities that are ecologically feasible, economically viable and socially acceptable. Sustainability is therefore based on three key elements: social well-being, economic growth, and environmental resource conservation. Sustainable socioeconomic activities improve the overall well-being of societies by protecting human and environmental health while creating economic opportunities. This has a positive long-term impact on individuals’ quality of life (QOL). Consequently, QOL and sustainability are intrinsically linked. That is, our relationship with the socioecological system determines the QOL that is achievable.

A diverse set of factors interact with shape QOL in a personal and the broader social context. The level of influence of each factor is determined by the needs of the individual or society. The higher the priority of the need, the greater the impact on well-being is perceived to be. For example, it is reasonable to assume that a biological need, such as food, is a priority need. If this need is not met, problems arise in a wide range of areas, ranging from public health to economic production. In this context, the impact of food security on social welfare plays a critical role in shaping both social welfare and perceived quality of life. This line of reasoning is confirmed by the fact that hunger is a priority item in the Sustainable Development Goals (SDGs) declared by the United Nations (UN). SDG 2 aims to end hunger worldwide, achieve food security, and promote sustainable agriculture [1], all of which ultimately relate to long-term QOL. Although food security is essential for achieving a desirable QOL, it must be understood as part of a broader system of interacting factors that collectively shape well-being. That is, QOL is an emergent property derived from a complex of interacting variables. In one sense, food security is necessary but not sufficient for achieving a desirable QOL. At this stage, the following research question comes to mind: “What is the importance of food security for sustainable and long-term QOL and what is its contribution to QOL compared to other components in the context of quality of life?”

Without a clear understanding of the key drivers of QOL, social interventions may miss their target, leading to resource inefficiencies and limited progress toward sustainable development goals. The main objective of this study is to contribute to the development of more effective social policies and intervention strategies by identifying the components of QOL in the context of low-income, underserved communities in Guilford County, North Carolina, and analyzing their contributions to the QOL of individuals. This study focuses on determining the ranking of the QOL value components, the relative contribution of food security and identifying demographic segments based on their disposition toward the QOL value components.

In the literature, there is ongoing debate on the precise definition of QOL [2,3,4,5]. This study draws on the definition proposed by the World Health Organization, which is “an individual’s perception of their position in life relative to their goals, expectations and standards as these are assessed within the context of their culture and value systems in which they live” [6]. Standard indicators of quality of life include wealth, employment, the environment, physical and mental health, education, recreation and leisure time, social belonging, religious beliefs, safety, security, and freedom. QOL is defined in a wide range of contexts, including the fields of international development, health care, economics, politics, and employment. For example, health-related QOL (HRQOL) is an evaluation of QOL and its relationship with health [7]. When assessing the role of food security in QOL in underserved communities, it is essential to comprehend the dimensionality of QOL from the perspective of the lived experience of the community.

This study investigates QOL through the lens of lived experience of residents in underserved, low-income communities. Improving quality of life (QOL) has become a key objective for international, national, state, and local governing bodies. QOL is a multidimensional concept that includes both objective and subjective dimensions: objective QOL refers to measurable indicators like income, health care, and access to services, while subjective QOL reflects individuals’ perceptions and satisfaction with these aspects of life [8,9].

Another perspective classifies individual quality of life based on two dimensions: chances/outcomes and internal/external [10,11,12]. This classification is particularly useful as it distinguishes four QOL dimensions based on external/internal typology. For example, the external typology includes livability and the utility of life afforded to the individual in the referenced environment, while the internal environment describes the life-ability and satisfaction of the individual with life in the referenced environment. Livability describes the external environment that shapes one’s opportunities to pursue a better quality of life. It is the focus of economic indices and social indicators that seek to measure QOL; it is generally referred to as standard of living or well-being [11,12,13,14]. The other individual kind of QOL is life-ability. It identifies the individual’s capacity to marshal their abilities and skills to seek and achieve a higher standard of living or personal well-being. Specifically, it includes an individual’s health status, education level, intellectual capacity, and social skills [8]. The life-ability aspect of the individual quality of life is the focus of the UN human development and poverty indices. The third aspect of an individual’s quality of life highlights their behavior in relation to others, their contribution to society, and the happiness of those around them. The fourth aspect, life satisfaction, emerges from the interaction between life-ability and environmental livability. In practical terms, this means the ability of the individual to exploit opportunities available in or provided by the environment to achieve a level of well-being that brings them satisfaction.

A comprehensive review of the literature on QOL underscores the pivotal roles of environmental factors and individual capabilities in shaping well-being. The environment in which an individual resides, coupled with their capacity to utilize their abilities to acquire resources, significantly impacts their standard of living and that of society and individuals within their immediate and extended circles. Yet, in measuring quality of life, emphasis is mostly on aggregate measures such as Gross Domestic Product (GDP), which leads to a popular false notion that QOL can be captured by a single measure. From the above discussion, it is also clear that the QOL of the individual depends on two factors. First, the ability and skill of the individual to make use of opportunities and resources in the environment to advance their quality of life. Second, the amount and quality of resources and opportunities available in the environment.

If we hypothesize that QOL is life’s goal, then individuals are naturally motivated to pursue actions that they believe will lead to a better QOL. In this paper, we define the series of actions that the individual takes, which over time falls into a stable pattern of preferred behavior, as the individual’s lifestyle. Thus, it is reasonable to conclude that the goal of a particular lifestyle is the attainment of a satisfactory QOL. In this light, one prominent academic view holds that preferences are a part of who we are and are stable (considered and rational), not easily swayed by external influences such as exhortations from teachers or pastors or pedantic lessons from experts on the benefits of modifying one’s preferences to achieve desired goals [15].

Still, a more recent academic view is that where one lives, the people one associates with, culture, history, the community as a whole, and many other external factors help to shape our preferences [16] and, as such, our lifestyle. This latter perspective supports the contention that lifestyle, as a pattern of behavior, is modifiable and provides strong justification for undertaking programs that target the modification of lifestyle to promote valued health and quality of life outcomes. On the other hand, the view that preferences are considered, coherent, and rational indicates that “experts” should not patronize or take for granted that people’s preferences are whimsical and devoid of a rational basis. Both perspectives offer practical insight into designing interventions that respect existing behaviors while promoting healthier lifestyles within a socioecological context. For example, based on the latter view, peoples’ lived experiences are shaped by their context. People learn from and adapt to their environment by developing strategies to survive and thrive. The first perspective helps us to understand that these strategies are considered, thought out, and coherent and not the product of whimsy. Efforts to address pressing social issues, including building a resilient food system, must first consider how context shapes peoples’ lived experiences and existing lifestyles before proposing solutions for advancing QOL in underserved communities. This means that success in our efforts is more likely if we start where people are and gradually move them to where we want them to be. Second, we must recognize that people and their lifestyles are embedded in a system shaped by multiple interacting factors [17]. Consequently, in building a resilient food system that advances QOL, it is crucial to understand the relative contributions of factors in the QOL value matrix and the pattern of segments that emerges from residents’ priority rankings.

To use the topical issue of addressing food insecurity as an example again, it should be recognized that food security is only one factor among several that impact lifestyle and overall quality of life for people living in insecure food communities. Addressing food insecurity in isolation may dampen the potential positive impact on quality of life if notice is not taken of how it fits into the overall lived experience or lifestyle of the target community. That is, providing food alone may not improve the QOL outlook for communities if we fail to acknowledge that communities and individuals are part of a larger system that defines their lifestyles and QOL statuses. Since we live in a socioecological system, our lifestyle determines how we relate to the environment, which, in turn, defines the level of resilience and sustainability that is achievable.

The identification and integration of complementary factors that could increase the effectiveness of strategies aimed at strengthening the food system’s resilience, eliminating food insecurity, and promoting healthier lifestyles is a potential avenue for future research. These complementarities have the potential to increase collective impact and help to achieve common QOL goals [18]. However, if these factors are not given adequate consideration, they have the potential to impede or even prevent the success of these endeavors. Consequently, the primary objective of this study is to determine the priority rankings of the components of the QOL value matrix, the contribution of food security to QOL and identify the pattern of the demographic segments that emerge from priority rankings of QOL components in low-income Guilford County communities. This study employed the Best–Worst Scaling (BWS) and the ISAC analytical framework to analyze the data. The ability to ascertain the contributions of food security and other components to QOL is instrumental in the development of sustainable and resilient food systems that are in line with the priorities established by these communities. This approach aligns with the mounting demand for place-based evidence, which is expected to inform the development of culturally sensitive and environmentally sustainable food systems, particularly in underserved communities within the United States.

## 2. Methodology

### 2.1. Research Design

In defining a structural QOL model, the initial model that comes to mind is that utilized by the WHO. According to this model, QOL is comprised of six components: physical health, psychological health, level of independence, social relationships, environment, and spiritual well-being [6,19]. In other seminal studies, dimensions such as food security, economic assets, and community assets have been added to these variables [20,21,22]. According to our research design, we first conducted in-depth interviews to identify the components of the community’s quality of life model. The components that we obtained were consistent with the previous literature. Since our study focus was on low-income communities within Guilford County, North Carolina, the United States, we endeavored to keep the structural model as simple and understandable as possible.

**Figure 1:**
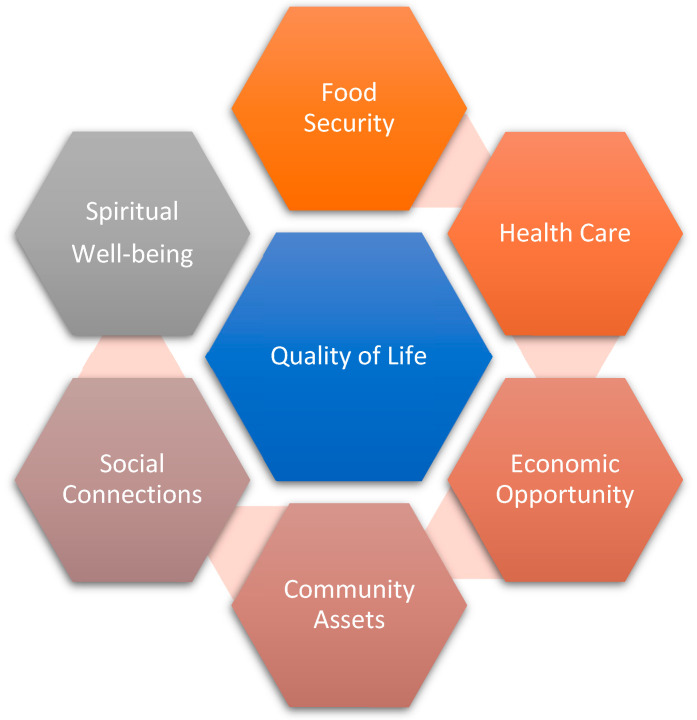
Structural model of quality of life.

Considering the data gathered from participants and the existing literature, this study identified six key factors comprising the model shown in Figure 1. These variables are postulated to impact quality of life (Figure 1).

### 2.2. Sampling and Data Collection

A structured telephone survey was developed to collect quantitative data. The questionnaire incorporated the identified factors using a Best–Worst Scaling (BWS) format, which was administered through the Qualtrics online platform. The sample size for the survey was determined using Nelson’s proportional sample size formula, based on a 95% confidence level and a 6% margin of error [23]. The primary population targeted (sample frame) in this study consisted of residents of low-income communities in Guilford County, North Carolina, where income and education levels are significantly lower than in other areas based on tract-level census indicators. Participants were recruited at random from a list of landline and cell phone numbers belonging to these neighborhoods, which were purchased from Dynata LLC, 4 Research Drive, Suite 300 Shelton, CT, 06484, the United States of America. Eligibility criteria included being an “adult” (age 18+), “residing in the target area”, and providing “verbal consent” to participate. Approximately three thousand calls were made, and 280 surveys were completed.

Although the task was simple in design, it often required multiple attempts to ensure clarity and accurate responses. Survey responses were recorded in a secure online database. The dataset was reviewed for quality control, and incomplete or logically inconsistent responses—such as contradictory selections—were removed to maintain the integrity and reliability of the analysis [23,24]. Ultimately, 213 valid responses were included in the final analysis.

Removing inconsistent questionnaires from the dataset is a crucial step to improve the quality of the survey data and ensure the reliability of the results [25]. Inconsistencies may result from contradictions, denials or illogical responses. Eliminating such data contributes to a more solid foundation for analysis. A prior study conducted at the University of North Carolina emphasized that removing inconsistent or misleading data from an analysis can increase the reliability of the results [26,27]. We subjected our data to this refinement process [28]. By removing these data, the sample size decreased to 213. So, the margin of error in the representativeness of the sample to the population increased slightly but was still within acceptable limits.

### 2.3. Data Analysis

The **ISAC Analysis Procedure** developed by Cankurt and Thomas was used to identify the preferences and characteristics of subgroups according to the objectives of this study [27]. The ISAC Analysis Procedure is a multi-stage analytical approach used to systematically examine and interpret complex phenomena, especially in the context of social sciences, consumer behavior, or any field where understanding underlying patterns and relationships is crucial.

Besides the ISAC Procedure, the main analysis method used in this study was Best–Worst Scaling (BWS). BWS was preferred by the researchers as it requires fewer comparisons compared to other alternatives and, thus, facilitates difficult survey administration [29,30,31]. In this study, each respondent was presented with a single Best–Worst Scaling (BWS) task containing all six attributes simultaneously (fixed full profile, single-task design). Respondents were asked to select the most and the least important attribute (i.e., best and worst) from the full set. It was used to analyze the data obtained via the telephone survey. In addition, simple descriptive statistics such as frequency, percentage, mean, and median were calculated to provide more detailed information about the population.

### 2.4. ISAC Analysis Procedure

The ISAC Analysis Procedure is a new multi-stage analytical approach that is utilized to systematically study and interpret complex phenomena, particularly in the context of social sciences [27]. The ISAC procedure was defined and developed with the objective of making the process more recognizable, easier to understand and apply. The ISAC Analysis Procedure has three stages:

**Stage 1. Identification:** The initial stage of the ISAC process is identification. At this stage, the ISAC Analysis Procedure serves as a diagnostic snapshot of the population by capturing the structure, distribution, and key demographic and contextual variables relevant to the research. The first step involves calculating descriptive statistics that reveal the structure of the data. These include means, standard deviations, minimum and maximum values, and frequencies for variables of interest. Following this, dimension reduction is conducted to identify variables for use in further analysis, with the aim of achieving the research purpose [32].

**Stage 2. Segmentation:** The second stage of the ISAC procedure is segmentation. This stage involves dividing a population, community, or target group into defined subgroups based on specific characteristics [33,34]. These characteristics may be demographic, psychographic, behavioral, geographic, or other variables related to the research focus [35]. The process of segmentation enables researchers to gain insight into the specific needs, behaviors, attitudes, and opinions of distinct groups (segments), thereby facilitating the development of strategies that are tailored to the unique requirements of each segment [27].

**Stage 3. Characterization:** The characterization stage represents the final and most pivotal phases of the ISAC Analysis Process. In this stage, the groups delineated in the segmentation process are subjected to a comprehensive examination, with the objective of elucidating the distinctive attributes of each segment [33]. In the characterization process, each segment is initially examined through the lens of pertinent data, including demographic information, psychographic characteristics, and behavioral tendencies [36]. Because of this analysis, the distinctive characteristics of each segment are discerned. Subsequently, fictitious characters, designated as “personas,” are constructed based on the segments. Personas represent a typical member of the segment and reflect the typical characteristics of that segment, including needs, motivations, and behaviors. Personas facilitate a deeper comprehension of the target audience and serve as a compass for the formulation of strategies and policies tailored to these personas [37,38].

In conclusion, the ISAC Analysis Procedure is particularly advantageous in domains such as marketing, as evidenced by its efficacy in this study, where it enables researchers and analysts to derive actionable insights through a systematic and structured examination of the data. The segmentation and characterization processes, in this case, enhance policymaking effectiveness by facilitating the customization of programs.

### 2.5. Best–Worst Scaling

BW Scaling was developed by Louviere and Woodworth (1990) as a multiple-criteria method for pairwise comparisons [39]. However, two years later, in 1992, a paper published by Louviere et al. demonstrated the scientific basis and application of this approach [39,40]. BWS is a method that employs the pairwise comparison of multiple options by asking respondents to select both the most and least attractive options or attributes from a set of options. It is an easy-to-apply method for the studying, ranking, and modeling of preference and choice problems for academics and practitioners in business, especially marketing, and the social sciences in general. BWS avoids many rating scale problems and appeals to those who want to measure subjective quantities with known measurement properties that can be easily interpreted and applied [29,41].

Best–Worst Scaling (BWS) is also known as maximum difference scaling. This method has emerged as a more robust alternative to the problems of bias, inconsistency, central tendency bias, and social desirability bias inherent in traditional ratings. By focusing on extremes, BWS enables researchers to obtain richer and more nuanced preference data and often achieve greater reliability than traditional methods [42]. It allows researchers to rank preferences clearly, providing insights into the relative importance of items [43,44]. The strength of BWS lies in its ability to extract clear preference signals by focusing on extremes that typically reflect more conscious judgments. However, one question that may arise regarding the BWS method concerns the options that are not preferred between the best and worst options. As the number of individuals surveyed (n) increases, there is a slight tendency to select b or w due to different preferences arising from individual differences. Even if selected by a single individual, its relative position is statistically determined.

Its main advantage for researchers stems from its ability to provide interval-scale data from simple ranked responses, which makes it a first choice for generating priority rankings. By simplifying respondents’ tasks while increasing the granularity of the data collected, BWS is an easy-to-apply alternative to methods such as simple ranking, pairwise comparison, and fuzzy pairwise comparison.

## 3. Results and Discussion

As explained in the method section, the research design follows the ISAC Analysis Procedure: it includes the stages of Identification, Segmentation, and Characterization.

### 3.1. Identification

In this phase the sample is described according to demographic variables and their subgroups as follows: age, race, gender, education, relationship status, employment status, and priority ratings established. Table 1 shows the number and percentage distribution of these variables according to their subgroups.

The findings obtained from the sample in Table 1 are compared and interpreted with respect to Guilford County, NC state data, and US averages.

Gender: The sample has a higher proportion of female respondents (59.62%) compared to male respondents (40.39%). Guilford County and the US exhibit a slightly more balanced gender distribution, with females accounting for approximately 52% of the population [45,46].

Age: The sample is predominantly made up of adults (52.53%), followed by early seniors (20.28%) and seniors (19.82%), with a lower proportion of young adults (2.30%). In contrast, Guilford County has approximately 9% of its population between the ages of 18 and 24 and 16% are 65 years of age or older [45,46]. Nationally, the US population has a higher proportion of young adults at 21% and a smaller elderly population at 16% [45]. The under-representation of young adults limits the sample from fully reflecting the perspectives of this group.

Race: The racial composition is primarily White (53.00%) and Black or African American (40.60%), with smaller representations of American Indian or Alaska Native (1.40%). Guilford County’s racial demographics indicate a 56% White and 35% Black or African American population [46], aligning closely with the sample. Nationally, the proportions are 60% White and 13.6% Black [45]. The elevated representation of Black participants aligns more closely with Guilford County than national averages, highlighting the region’s unique demographic composition.

Education: Participants with a bachelor’s degree (35.00%) and a master’s degree or higher (27.20%) are prominent in the sample, collectively surpassing 60% of respondents. Comparatively, in Guilford County, approximately 30% of residents hold a bachelor’s degree and 14% hold graduate or professional degrees [47]. Nationally, these rates are 21% and 13%, respectively [45]. The sample reflects a higher education level than both the regional and national averages, suggesting a potentially more specialized or affluent respondent pool.

Relationship Status: Nearly half of the participants are married and live with their spouse (47.00%), while 21.20% are single. Guilford County reports a marriage rate of 43%, with 37% of adults being single [45]. Nationally, the marriage rate is 48%, with a similar proportion of singles [48]. It is observed that the calculated relationship values in the sample are close to the NC and national values.

Employment: The majority of respondents work full-time (61.30%), while 24.40% are retired. Guilford County’s labor force participation rate is 63%, closely reflecting the employment trend of this sample [47]. Nationally, the labor force participation rate is 62.6% [49]. The data obtained from the samples are close to the county, state and national values.

For priority ratings, the results indicated that spiritual well-being (0.23) emerged as the most significant factor in terms of relative weights, followed by health care (0.21), economic opportunity (0.16), and food security (0.07), while community assets (−0.24) and social connections (−0.42) were considered the least influential. This research provides valuable insights into the priorities that can enhance life quality, informing policymakers and stakeholders in targeted community planning and resource allocation. The findings of this study emphasize the pivotal role of spiritual well-being in enhancing quality of life within the community (Table 2).

One of the main tasks of policymakers, perhaps the first, is to improve the QOL of society. In doing so, it is essential for them to identify the most effective factors for using scarce public resources in the most efficient way. The insights from this study offer the opportunity to increase the efficiency of efforts to improve overall quality of life.

**Figure 2:**
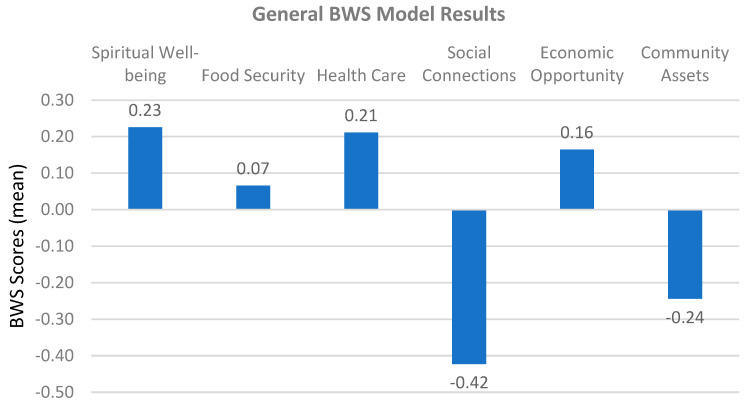
Relative weights of QOL factors.

The present analysis, based on the Best–Worst Scaling (BWS) methodology, assesses six key dimensions affecting quality of life under the general model: spiritual well-being, food security, health care, social connections, economic opportunities, and social assets. The relative impacts of these factors on overall quality of life are identified. Due to the nature of the BWS method, some factors received positive and some others negative values. However, the negative ones are not interpreted here as having a negative impact. This is a relative ranking, with positive factors having a higher impact and negative factors having a lower impact relative to zero value. The BWS method is basically a method of determining the relative weights of other factors based on the maximum difference between the most and least influential factors (Figure 2).

In this study, spiritual well-being was intentionally left open to the interpretation of participants to capture both religious and non-religious expressions of meaning, purpose, and inner peace. Among the factors assessed, the results underline the most important role of spiritual well-being. Spiritual well-being emerges as the most important positive contributor to QOL, with a weight of 0.23. This finding underscores the critical role of personal and community spiritual practices in supporting spiritual well-being and emotional stability. Moreover, this finding is consistent with studies emphasizing the importance of spiritual satisfaction in supporting psychological resilience and emotional stability [50]. Spiritual practices are often linked to improved mental health outcomes and increased life satisfaction, especially in communities experiencing distress [51].

Health care emerges as the second most influential factor, with a weight of 0.21. Access to quality health care has long been recognized as a cornerstone of quality of life, as highlighted by Marmot and Wilkinson (2012), who argue that equitable health systems play a crucial role in reducing social inequalities [52]. Studies have shown that improving health care access can reduce mortality rates and increase subjective well-being, particularly in vulnerable populations [53,54].

Economic Opportunities also contribute positively to quality of life but have a relatively higher weight with a value of 0.16. This reflects the idea that economic security provides a foundation for addressing other dimensions of well-being, such as access to education and social mobility [55]. The literature strongly supports the importance of economic opportunity in promoting a sense of independence and social inclusion, particularly in low-income communities [56]. Food security has a moderate contribution of 0.07, suggesting that its impact on quality of life may be overshadowed by other areas when basic nutritional needs are met. This observation is in line with the findings of Smith and Haddad [57], who find that food security is a critical determinant of well-being primarily in contexts of scarcity. However, the existing literature reinforces its critical importance in addressing health disparities and promoting physical health, particularly in low-income and marginalized populations [58,59].

At the least influential end of the spectrum is social connections, with a value of −0.42 Holt-Lunstad et al.’s [57] research illustrates the negative effects of social isolation, which is strongly associated with poor mental health outcomes and reduced life expectancy. The low contribution of community assets, with a value of −0.24, also highlights the detrimental effects of limited access to social resources and echoes Putnam’s (2000) [60] findings on the erosion of social capital and its effects on collective well-being. Evidence suggests that disparities in access to community assets, such as parks, transportation, and public facilities, exacerbate socioeconomic inequalities and hinder community well-being [61].

In summary, these findings reinforce the multidimensional nature of QOL determinants and demonstrate the interplay between individual, social, and systemic factors. They emphasize the need for policies that prioritize spiritual well-being, equitable health care, and strong economic opportunities while addressing social and societal deficiencies that undermine QOL. To enhance spiritual well-being in practice, initiatives such as community-based spiritual support programs, faith-based counseling, and opportunities for meaningful social engagement through local organizations can play a crucial role. Although food is a basic biological necessity, once this need is addressed at the most basic level or even when the long-term prospect of addressing the need is not guaranteed, other perceived QOL factors seem more salient and relevant to respondents in the sample. This phenomenon is not completely unheard of. The story of a Moroccan who prioritized the purchase of a television even though he was uncertain how he would provide for his next meal [16] illustrates this phenomenon. A resilient food system that removes the risk of hunger and malnutrition allows even resource-poor and underserved communities to pursue and realize the fulfillment of meaning in their lives.

### 3.2. Segmentation

After seeing the overall picture in the previous section, deeper insight is obtained through segmentation. In the segmentation stage, the preferences of the subgroups that make up the overall model are compared. Each subgroup represents a segment. We tried to determine the change in the preferences of the individuals participating in the survey according to these segments. In the following section, the general model will be compared with the subgroups of gender, age, race, education, and employment.

**Figure 3:**
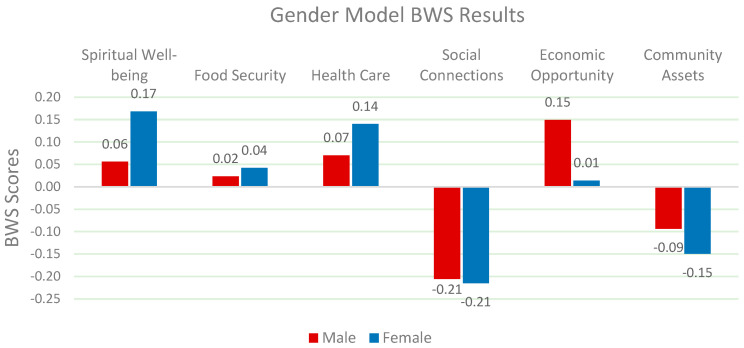
Relative weights of QOL factors by gender.

The analysis reveals clear significant differences in the determinants of QOL between genders (*Chi-Square: 16.925 p < 0.01*). Female participants prioritize spiritual well-being and health care, while male participants place more emphasis on economic opportunities. According to Koenig et al. (2012), spiritual satisfaction plays a greater role in enhancing emotional resilience and psychological stability for women [50]. On the other hand, Case & Deaton (2015) argue that men often view economic security and opportunity as the primary drivers of life satisfaction [53]. These findings suggest that policymakers should tailor their strategies to focus on health care and spiritual support for women and economic insecurity for men (Figure 3).

**Figure 4:**
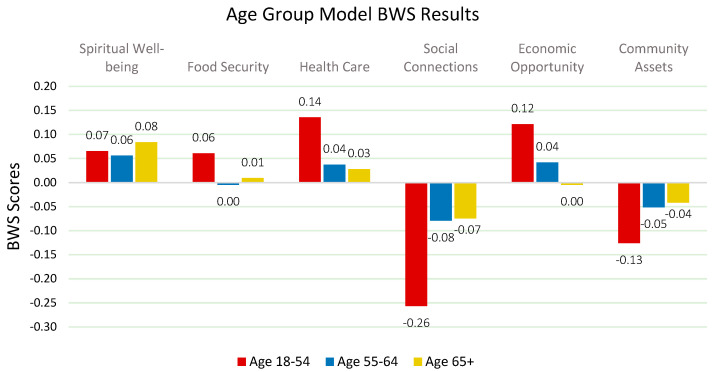
Relative weights of QOL factors by age group.

The analysis reveals expected differences between age groups (*Chi-square: 2.74 p < 0.10*). When determining age groups in the research design, young adults (18–24) were planned as a separate group. However, due to the small number of young adults in the survey results, they were combined with adults. Adults (18–54) prioritize health care and economic opportunities. In contrast, early seniors (55–64) and seniors (65+) emphasize spiritual well-being and health care as primary factors. This is consistent with Holt-Lunstad et al., who suggest that social connections are critical to the emotional well-being of younger adults, and VanderWeele et al., who highlight the importance of spiritual well-being in supporting resilience in older populations [51,57]. These age-related differences highlight the need for age-specific interventions to effectively address quality of life (Figure 4).

**Figure 5:**
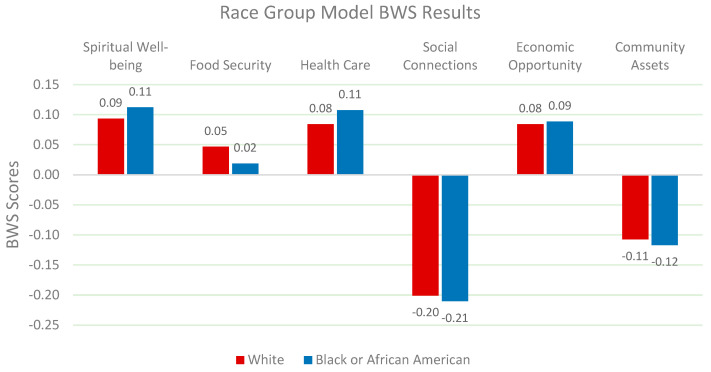
Relative weights of QOL factors by race.

Racial differences significantly influence the determinants of QOL. White participants prioritize health care and economic opportunities, while African American participants place greater importance on spiritual well-being, health care, and economic opportunity [62]. Wilkinson & Pickett (2010) argue that unequal access to health care exacerbates existing inequalities in well-being [61]. These findings suggest that policies must address structural disparities in health care and food security across racial groups (Figure 5).

**Figure 6:**
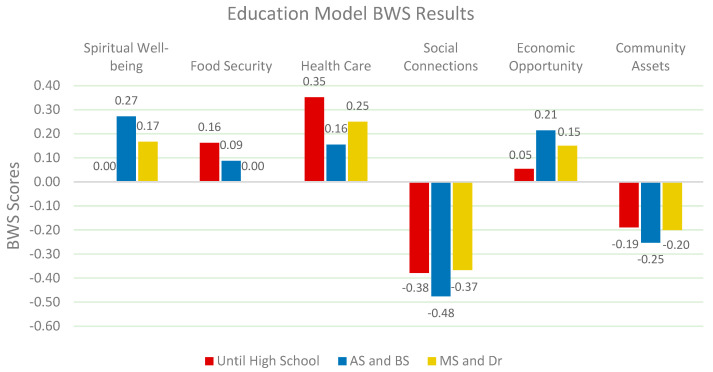
Relative weights of QOL factors by education.

Education level also shapes QOL priorities. Participants with a bachelor’s degree or higher prioritize economic opportunities and health care, while those with lower levels of education emphasize food security and community assets. According to Sen (2013), education serves as a key enabler for economic mobility and access to health care [55]. Meanwhile, Gundersen & Ziliak (2015) highlight the relationship between lower educational attainment and increased vulnerability to food insecurity [59]. This suggests that education-centered strategies can play a pivotal role in improving QOL outcomes across different segments (*Chi-square: 49.760 p < 0.05*) (Figure 6).

**Figure 7:**
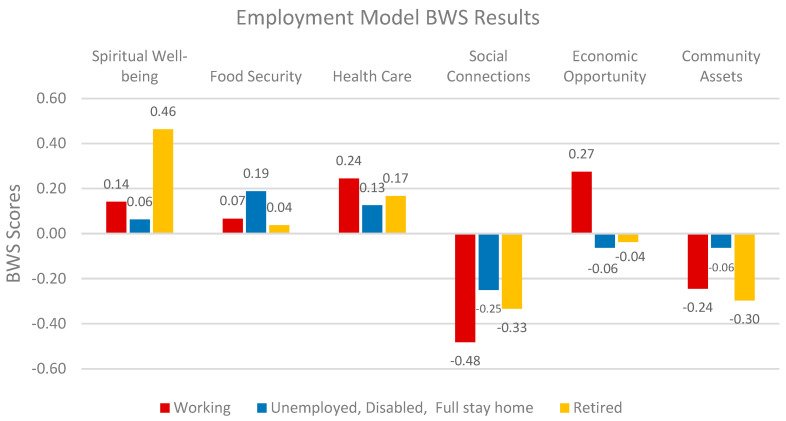
Relative weights of QOL factors by employment.

Employment status emerges as a key factor in QOL outcomes. Full-time employees prioritize economic opportunities and health care, while retirees focus on spiritual well-being and health care (*Chi-square: 46.502 p < 0.05*). In contrast, unemployed individuals place higher importance on food security and community assets. Case & Deaton (2015) argue that unemployment significantly reduces life satisfaction and overall well-being [53]. Similarly, Seligman et al. (2010) emphasizes the connection between unemployment and increased food insecurity risks [58]. These findings suggest that employment-focused strategies should address both economic stability and basic needs like food security (Figure 7).

**Figure 8:**
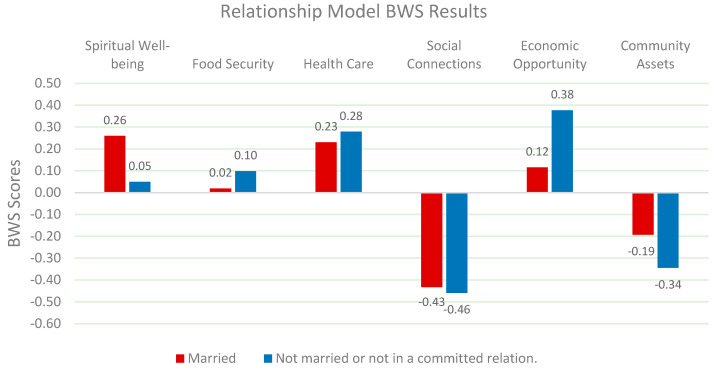
Relative weights of QOL factors by relationship.

Relationship status has a significant impact on perceptions of QOL (*Chi-square: 48.478 p < 0.05*). Married individuals prioritize spiritual well-being and health care, while single individuals value economic opportunities and social connections. Research by Holt-Lunstad et al. (2015) suggests that married individuals often benefit from stronger emotional and spiritual support networks [57]. While Putnam (2000) highlights the importance of social connections for single individuals in maintaining psychological well-being [60]. These findings suggest that relationship-specific interventions are essential for improving QOL outcomes (Figure 8).

Spiritual well-being is the most critical factor for older and retired people. Spiritual support and fulfillment especially strengthen emotional resilience and improves quality of life. Health care is a priority for middle-aged individuals and female respondents. Access to health services has a major impact on quality of life, especially for working-age individuals. Economic opportunity is a key factor for young adults and full-time workers. Economic security and career opportunities have a direct impact on perceptions of quality of life. Food security is an important factor for individuals with low levels of education and the unemployed. Problems with food access negatively affect the quality of life of these segments. Social assets mean that accessing social resources is essential for those on low incomes and those who are unemployed. While social connections are an important factor for young adults, they are less of a priority for older adults and retirees.

The segmentation analysis reveals substantial differences in QOL priorities across demographic groups. Gender, age, race, education, relationship status, and employment status each influence how individuals perceive and prioritize factors contributing to their well-being. Policymakers should develop targeted strategies that address these unique needs, focusing on health care, spiritual well-being, economic opportunities, food security, and social connections. By adopting a more nuanced and segmented approach, interventions can be more effective in improving the overall quality of life across diverse communities.

### 3.3. Characterization

Following the segmentation analyses, we generated persona groups based on different demographic groups’ preferences among QOL factors (spiritual well-being, food security, health care, etc.). Different personas were revealed, shaped by the priorities that they placed on key quality of life (QOL) factors. This characterization analysis provides a clear picture of how factors affecting the quality of life of different demographic groups vary. Variables such as age, gender, education, employment status, and marital status shape personal priorities and reveal different needs that policymakers need to address. The personas that are identified by the results of analysis are shown below.

Spiritual Well-being Focused Retired Female: The most important determinant of quality of life for *educated*, *retired*, *married*, *and female persona* is spiritual well-being. While this group places spiritual support and peace of mind at the center of their quality of life, health care stands out as another important factor. Social connections and economic opportunities have a relatively lower priority for this group compared to other factors. This persona has indicated that food security is not a priority. This situation may imply that individuals typified by this persona have a regular income from a pension. Additionally, food assistance programs or community-based resources (e.g., primarily church donations and church sponsored neighborhood support) may be available. However, the fact that this group does not prioritize food security may mean they perceive the church and their spirituality as instrumental in meeting their food security needs. 

Economic Opportunity Seeking Working Single Male: Economic opportunities and career development are the most important factors determining quality of life for *young and middle-aged*, *full-time employed*, *single males with a college or bachelor’s degree*. While these individuals prioritize financial security and job opportunities, health care also stands out as an important element of support. A possible reason why this persona views food security as a low priority may be that their current economic situation allows them to make ends meet for food security. Access to food is not generally seen as a threat for individuals who work full-time and are usually single. However, at this point, we must consider “hidden food insecurity”.

Health Care Focused Middle-Aged and Young Women: For *young and middle-aged*, *working*, *single*, *female individuals with a high school diploma or less*, health care is the most influential factor in quality of life. For this group, easy access to health services and quality health infrastructure makes the biggest contribution to their quality of life. Social connections and economic opportunities take a back seat. Food security, while not taking precedence over economic opportunities and social connections, is nonetheless an important factor that cannot be ignored for this persona. The reason for this is that while middle-aged working women may consider food security important for their children’s nutrition, their employment may alleviate this concern and push food security down the list of priorities.

Food Security Focused Unemployed Singles: Food security is the most fundamental determinant of quality of life for people who are *unemployed*, *single*, *and have low levels of education*. Food security and meeting basic needs are critical for this group. Individuals who prioritize food security over other quality of life values in society are unemployed, low-educated, young, and middle-aged individuals who do not value social relationships.

In sum, the personas derived from segmentation highlight the multidimensional and context-specific nature of quality-of-life priorities. Effective policy interventions should consider these nuanced personas, such as addressing the mental and health needs of older adults, increasing food security for vulnerable groups, and promoting economic opportunities for younger and working-age adults. This persona-based approach ensures that resources are allocated efficiently and equitably to meet the most pressing needs in each demographic segment. Designing policies and programs that consider the specific needs of these different persona groups will provide more effective and sustainable solutions to improve quality of life. These findings imply that sustainable food systems cannot be designed as one-size-fits-all solutions. Instead, it is essential that these initiatives be customized to align with the distinct priorities of diverse demographic groups, particularly those for whom food security is of paramount importance in determining their quality of life.

## 4. Conclusions

This study provides a detailed analysis of the factors affecting quality of life (QOL) and provides comprehensive findings with the potential for helping policymakers to use resources more effectively. Using the Best–Worst Scaling (BWS) methodology and the ISAC (Identification, Segmentation, and Characterization) analysis procedure, we identified six key factors influencing QOL: food security, spiritual well-being, health care, economic opportunity, community assets, and social connections.

This study highlights the differences in the QOL priorities of different demographic groups and suggests that policy recommendations should consider these differences. These findings contribute to the development of sustainable and resilient food systems by highlighting the specific quality of life priorities of underserved populations, which should be addressed in an integrated and culturally responsive manner. Spiritual well-being, health care, economic opportunities, and food security are the key factors highlighted in this study. Below, the persona-based policy recommendations identified by these findings are detailed. Integrating spiritual well-being, access to health care, and economic opportunities alongside food access strategies will enhance the sustainability and resilience of community-level food systems.

For educated, married, and retired women, spiritual well-being stands out as the most important determinant of quality of life. This group prioritizes spiritual support and peace of mind for improving quality of life. Access to health care is also a priority factor for this group. In line with these findings, it is important to leverage the role of the church in supporting food security while facilitating spiritual support groups and spiritual activities. In addition, solutions such as mobile health centers that facilitate access to health care for the elderly may also be among effective policies for this group. Such programs can reduce health expenditures and improve overall quality of life by reducing feelings of loneliness.

Economic opportunities are the most important determinant of quality of life for young and middle-aged working single men. These individuals prioritize economic security and career development. For this group, the findings suggest that it is critical to scale up career development programs, increase entrepreneurship support funds, and provide incentives that strengthen the local labor market to enhance QOL. In addition, improving the skills of this group through technological and vocational training programs can increase job satisfaction and social inclusion.

Among young and middle-aged women, access to health care is one of the factors most affecting quality of life. Facilitating access to health care services for this group of low-educated, employed or unemployed women is of great importance. Expanding women-specific health programs, introducing mobile health centers, and expanding health insurance are among the recommendations for this group. Such policies can support societal labor productivity while improving the quality of life of individuals.

Although food security was ranked fourth in terms of overall importance, it emerged as the top concern among vulnerable groups, particularly the unemployed, low-educated, and single. This highlights the need for targeted interventions to enhance QOL f for these individuals. To meet the basic needs of this group, there is a need to expand options that address food security. For example, expanding the reach and coverage of food assistance programs, community gardens and cooperative projects to support local food production. Addressing food security through these local initiatives creates options, diversifies the food system enhance access builds resilience and supports environmentally sustainable food production practices. In addition, increasing the logistical capacity of food banks and facilitating the provision of low-cost or free food can also be among effective policies for this group. Of course, policymakers and program managers will act on these findings within the context of organization capacity and available resources.

Overall, the findings indicate that although food security is a component of QOL, it lags spiritual wellness, health care, and economic opportunity in terms of contribution to QOL but is rated above community assets and social connectedness. We infer from our in-depth interviews that food security’s role in QOL is to provide a cushion against risk of hunger and malnutrition allowing even underserved resource-poor individuals to pursue meaning and fulfilment that is made possible by realizing spiritual well-being, health, and economic opportunity. Another consideration is that spiritual well-being is a proxy for social connectedness in underserved communities where the church and its activities provide opportunities for meaningful social interaction, comradery, and support with greater significance and meaning than everyday social interactions. Pink [63] observed that in times of plenty, with the abundance and availability of material things, individuals seek relevance and meaning for the fulfillment of QOL. In this context, a secure and resilient food system plays a pivotal role in ensuring that individuals are food-secure and are thus free to pursue meaning that transcends material objectives.

Additionally, this study demonstrates the effectiveness of segmentation and persona-based approaches in policymaking. Policymakers should develop solutions that consider the different priorities and needs of each demographic group, act strategically in resource allocation, and implement targeted programs at the local level, which will pave the way for sustainable and effective social policies. This study provides a roadmap for the more efficient use of limited resources to meet the most critical needs. Accordingly, prioritizing factors such as spiritual well-being, economic opportunities, health care, and food security will be an important step in increasing social welfare.

### Limitations

This study did not model intersectional effects (e.g., race × gender) due to the sample size and the exploratory nature of the segmentation. Future studies should consider examining such interactions. The sample slightly over-represents older, female, and highly educated individuals, which may limit the generalizability of subgroup-specific findings.

## Figures and Tables

**Table 1:** The values of demographic variables by subgroups.

Variables	Categories	Count	%
Gender			
	Female	124	59.62
	Male	84	40.39
Age			
	Young Adults (18–24)	5	2.30
	Adults (25–54)	114	52.53
	Early Seniors (55–64)	44	20.28
	Seniors (65+)	43	19.82
	PNR or missing *	11	5.07
Race			
	American Indian or Alaska Native	3	1.40
	Black or African American	88	40.60
	White	115	53.00
	PNR or missing *	11	5.10
Education			
	Master’s degree or higher	59	27.20
	Bachelor’s degree	76	35.00
	Associate’s degree	25	11.50
	Some college but did not complete degree	26	12.00
	High school graduate or GED completed	9	4.10
	Some high school	1	0.50
	PNR or missing *	21	9.70
Relationship			
	Married and living with spouse	102	47.00
	Married and not living with spouse	17	7.80
	Not in a committed relationship (i.e., single)	46	21.20
	Not married but in a committed relationship	11	5.10
	Not sure	3	1.40
	PNR or missing *	28	12.90
Employment			
	Working full time, 35 h/week or more **	133	61.30
	Working part-time, less than 35 h/week	7	3.20
	Retired	53	24.40
	A full-time stay-at-home parent	8	3.70
	Unemployed	6	2.80
	Disabled, not able to work	1	0.50
	PNR or missing *	9	4.10

* Prefer not to respond or missing value; ** includes self-employed.

**Table 2:** Best–Worst Scaling results.

Variables	Best Frequency	Worst Frequency	B-W	Mean (B-W)	Normalized Relative Weights (%)
Spiritual Well-being	70	22	48	0.225	25.56
Food Security	22	8	14	0.066	19.26
Health Care	52	7	45	0.207	25.00
Social Connections	10	100	−90	−0.423	0.00
Economic Opportunity	53	18	35	0.164	23.15
Community Assets	6	58	−52	−0.239	7.04

Friedman test statistic: 24.11 (*p* = 0.00); n = 213.

## Data Availability

The original contributions presented in this study are included in this article; further inquiries can be directed to the corresponding author.
